# Superiority of Surgical Treatment in Stage 2 Acute Empyema: A Perspective on Lung Volume Improvement

**DOI:** 10.7759/cureus.83856

**Published:** 2025-05-10

**Authors:** Hiroyasu Matsuoka, Hirochika Matsubara, Mio Ota, Hiroyuki Nakajima

**Affiliations:** 1 Surgery, Kofu Municipal Hospital, Kofu, JPN; 2 Thoracic Surgery, University of Yamanashi, Kofu, JPN

**Keywords:** drainage, empyema, lung volume, stage 2, surgical treatment

## Abstract

Introduction: Early surgery for acute empyema can shorten hospitalization and reduce complications, especially in Stage 2 cases. Inflammation and fibrosis can impair long-term pulmonary function, but few studies have evaluated post-treatment lung recovery. We hypothesized that surgery promotes better lung re-expansion than drainage alone. To test this hypothesis, we analyzed lung volume changes on computed tomography (CT) scans before and after treatment.

Methods: This retrospective, single-center study included adult patients with Stage 2 acute empyema treated from 2012 to 2024 at Kofu Municipal Hospital, Kofu, Japan. Patients were included if they had CT scans before treatment and at least one month after discharge. CT-based lung volumes were analyzed using SYNAPSE VINCENT^®^ (FUJIFILM Corporation, Tokyo, Japan) software. Since healthy-state CT scans are rarely available, the original volume of the diseased lung was estimated using the volume of the contralateral healthy lung, assuming a right-to-left volume ratio of 55:45. The primary outcome was the lung improvement rate on post-treatment CT. Secondary outcomes included hospital stay, drainage duration, and antibiotic use. Propensity score matching was used to adjust for baseline differences.

Results: Among 131 Stage 2 empyema cases, 25 met the inclusion criteria (10 surgery and 15 drainage). Surgery was performed after a median of seven days due to poor drainage. The surgery group showed significantly better lung improvement (82% vs. 68%, p < 0.01). Hospital stay and drainage duration were similar, but antibiotic use tended to be longer in the drainage group. After matching (nine patients per group), lung improvement remained superior in the surgery group (79% vs. 66%, p = 0.012). A statistically significant time-dependent improvement in total lung volume was observed in the surgery group following treatment (p = 0.04).

Discussion: Surgical intervention led to better lung volume recovery than drainage alone. However, selection bias may exist, as surgery was often delayed until after drainage failure. In three cases, limited decortication may have reduced recovery. This study only included patients with follow-up CT scans and good outcomes, potentially underestimating the full impact of treatment. Early surgical intervention may provide greater benefits by preventing disease progression and facilitating lung re-expansion.

Conclusion: Surgery for Stage 2 acute empyema significantly improves post-treatment lung volume recovery. Early surgical intervention should be considered in appropriate cases to optimize pulmonary outcomes.

## Introduction

Acute empyema is an infectious condition characterized by the accumulation of purulent pleural effusion, often secondary to pneumonia. Effective infection control requires adequate drainage of the pleural cavity. However, in advanced stages, fibrin deposition and the formation of thickened septations can impede percutaneous drainage, necessitating surgical intervention [1].

According to the classification by the American Thoracic Society, pleural empyema progresses through three stages: Stage 1 is defined by a free-flowing, non-loculated effusion; Stage 2 involves the development of fibrinous septations and purulent fluid, often referred to as a complicated effusion; and Stage 3 is characterized by fibrous pleural thickening, leading to lung entrapment [1]. Early surgical intervention in acute empyema shortens hospital stays and reduces treatment failure and complication rates [2-4]. In particular, for Stage 2 acute empyema, randomized controlled trials have demonstrated that video-assisted thoracoscopic surgery (VATS) is superior to chest tube drainage alone [4-6].

Although several clinical guidelines for empyema exist, no clear consensus has been reached regarding optimal management of Stage 1 disease. However, for Stage 2 empyema, early surgical intervention is widely recommended [7-9].

In terms of the impact of empyema treatment on respiratory function, previous studies on chronic empyema - often corresponding to Stage 3 - have reported improvements in parameters such as percent forced vital capacity (%FVC) following surgical decortication [10,11]. However, to the best of our knowledge, no studies have reported on this apart from CT improvement, which is a tool used to assess the improvement of respiratory function.

Unlike chronic empyema, patients with acute empyema are often in an unstable condition, with reduced performance status (PS) due to systemic inflammation and metabolic exhaustion [12]. This clinical instability makes accurate assessment of baseline pulmonary function difficult, thus complicating the prediction of post-treatment differences. This may explain the lack of published data addressing this issue.

However, total lung volume calculated from computed tomography (CT) imaging correlates strongly with %FVC [13]. Based on this, we considered using CT-derived lung volume as a surrogate for evaluating changes in respiratory function before and after treatment for empyema. We also sought to assess the degree of lung re-expansion following treatment for acute empyema using CT-based analysis.

However, in evaluating re-expansion, a simple comparison of the affected lung volume before and after treatment may be misleading, as more severely collapsed lungs may appear to improve more dramatically. Ideally, the extent of lung recovery would be assessed by comparing CT-derived lung volumes from a healthy pre-disease state and after treatment. In reality, however, pre-illness CT scans are rarely available in clinical practice.

To address this limitation, we propose an alternative approach: estimating the original lung volume on the affected side using the volume of the contralateral lung. Although both lungs can be affected simultaneously, involvement is often asymmetric. Previous studies suggest that normal lung volume distribution is approximately 45:55, with the right lung being larger [14]. Using this ratio, we estimated the expected lung volume on the affected side before disease onset and compared it to the actual post-treatment volume measured on CT. This allowed us to calculate the percentage of lung volume recovery after treatment.

In this study, we applied these two methods of evaluation to determine whether surgical intervention offers superior outcomes in terms of respiratory function and lung volume recovery, compared with chest drainage alone. The objective of this study is to evaluate and compare the efficacy of surgical intervention and intercostal drainage in terms of lung recovery and associated clinical outcomes in adult patients diagnosed with Stage 2 empyema.

## Materials and methods

Patients

This is a retrospective, single-center, case-control observational study of patients who received thoracic drainage or surgical treatment for Stage 2 acute empyema at Kofu Municipal Hospital, Kofu, Japan, from 2012 to 2024 (approval no. R6-11). Patients aged 18 years or older who were hospitalized and treated for acute empyema and pleuritis were selected from the electronic medical record. Among the selected patients, those whose CT scans were taken both before treatment and at least one month after discharge were included.

Acute empyema was diagnosed as the presence of pleural thickening on pre-treatment CT and a pleural effusion that was grossly purulent or an exudative effusion on biochemical examination. Cases in which all pleural effusions were drained after the first chest cavity drainage were excluded, as these were considered Stage 1 empyema. Patients with abnormalities in pre- or post-treatment CT images that caused a reduction in lung volume in the healthy lungs were also excluded from the study. Patients who underwent surgical intervention under general anesthesia during the course of treatment were assigned to the surgery group, while those who received only percutaneous pleural drainage were classified into the drainage group.

Treatment procedures

Patients were referred to the Department of Respiratory Medicine with a diagnosis of acute empyema or pneumonia. When pleural effusion was identified at the initial visit or during pneumonia treatment, acute empyema was diagnosed based on imaging findings and/or pleural fluid analysis. Once acute empyema was confirmed, the feasibility of chest drainage was assessed by the respiratory physicians and performed when deemed necessary. Antimicrobial therapy was initiated with broad-spectrum antibiotics at the start of treatment and was subsequently adjusted based on the identification of causative organisms. Percutaneous pleural drainage was performed after confirming the presence of empyema using CT and ultrasound. A 20Fr or 22Fr trocar catheter was typically inserted under local anesthesia through the lateral chest wall. The drain was removed at the discretion of the attending physician when drainage was less than 100 mL per day. In cases of inadequate drainage, the attending physician decided whether to initiate additional drainage or refer the patient to surgery. Surgical intervention involved placing an initial port directly above the empyema cavity or the drainage insertion site, followed by the addition of up to two additional ports via thoracoscopic surgery after adhesiolysis, or a single-port approach. If lung repair was required or adhesiolysis was not feasible, thoracotomy was performed. In all cases, debridement was first performed, and if lung expansion was poor, partial decortication was carried out at the discretion of the surgeon. Subsequently, one or two 20Fr or 22Fr drains were placed.

Outcome

The primary outcome was defined as lung volume improvement observed on post-treatment CT, assessed by the change in total lung volume from pre- to post-treatment and the improvement rate of the affected lung. The secondary outcomes included length of hospital stay, duration of total drainage, and duration of antimicrobial use. Outcomes were compared between the surgery and drainage groups (those who underwent surgery during the treatment course). Factors influencing treatment selection were compared between the groups, including age, sex, BMI, smoking index, the presence or absence of diabetes, PS ≥ 2, use of anticoagulation therapy, and pre-treatment lung collapse rate from CT images. The timing of CT imaging following treatment was not predetermined. Given that a longer post-treatment interval is generally associated with improved pulmonary recovery, this variable was incorporated into the analysis. Pre- and post-treatment CT images were analyzed using SYNAPSE VINCENT^®^ (FUJIFILM Corporation, Tokyo, Japan). Right, left, and total lung volumes were subsequently calculated.

Pre- and post-treatment CT images were analyzed using SYNAPSE VINCENT^®^ and right, left, and total lung volumes were calculated. The lung collapse rate before treatment was calculated as follows:

\[ \text{Lung collapse rate (%)} = \left( \frac{\text{Measured affected lung volume (before treatment)}}{\text{Predicted affected lung volume (before treatment)}} \right) \times 100 \]

**Figure 1:**
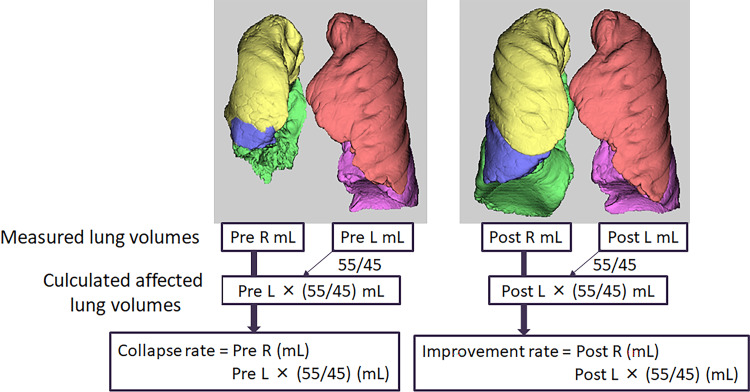
Calculation of collapse and improvement rates In cases of right-sided empyema, the predicted volume of the right lung was estimated by multiplying the measured volume of the contralateral (left) lung before treatment (Pre L, mL) by the standard right-to-left lung volume ratio (55:45). The lung collapse rate was then calculated by dividing the measured right lung volume before treatment (Pre R, mL) by this estimated value. Post-treatment lung recovery was assessed in the same manner: the predicted right lung volume was calculated by multiplying the post-treatment left lung volume (Post L, mL) by 55:45, and the lung improvement rate was determined by dividing the measured post-treatment right lung volume (Post R, mL) by this predicted value. For cases of left-sided empyema, the same method was applied, using the inverse ratio (45:55) to estimate the predicted volume of the affected left lung based on the contralateral (right) lung.

Improvement in lung volume after treatment was defined as the “lung improvement rate,” calculated using the post-treatment CT volumes as follows (Figure 1):

\[ \text{Lung improvement rate (%)} = \left( \frac{\text{Actual affected lung volume (post-treatment)}}{\text{Predicted affected lung volume (post-treatment)}} \right) \times 100 \]

Bias mitigation

The presence of bias in the decision to perform surgery was considered to exist. Therefore, we used propensity score matching based on factors that differed between the two groups in relation to treatment selection. To match cases, a logistic regression analysis was performed, using the presence or absence of surgery as the objective variable and the factors related to treatment selection (with p < 0.3) as explanatory variables to create a propensity score variable.

Sample size

The required sample size was calculated to be seven cases for each group, with a standard deviation of 10, an alpha error of 0.05, power = 0.8, and a 1:1 ratio for each group, assuming a mean difference in expected lung improvement rate of 15%. The expected lung volume improvement rate was set at 15%, based on previous reports indicating that pulmonary decortication for chronic empyema resulted in improvements in %FVC ranging from 15.66% to 29.7%, with 15% representing the lower bound of this range [10,11]. To perform propensity score matching in this study, a total of 28 cases - 14 in each group - were deemed necessary. Accordingly, case accumulation over a long period, from 2012 to 2024, was required to fulfill this number.

Statistical analyses

Categorical variables were compared using Fisher’s exact tests, and continuous variables were compared using nonparametric Mann-Whitney U-tests. Statistical significance was set at p < 0.05. The comparison of total lung volume pre- and post-treatment was conducted using repeated measures analysis of variance. All statistical analyses were performed using EZR^®^ (Saitama Medical Center, Jichi Medical University, Saitama, Japan), which is a graphical user interface for R^®^ (The R Foundation for Statistical Computing, Vienna, Austria), designed for biostatistical functions frequently used in clinical research. Using this propensity score, we extracted controls matched to the surgery group (drainage group = 1:1), with a caliper width of 0.2. The same statistical analysis was performed on the two extracted groups.

## Results

A total of 298 cases of empyema were treated. Among these, 131 cases were classified as Stage 2, with 25 meeting the inclusion criteria. Ten patients were assigned to the surgery group, and 15, to the drainage group. No significant differences were observed in patient backgrounds (Table 1).

**Table 1:** Characteristics of the two treatment groups (all cases) CT: computed tomography; SD: standard deviation Categorical variables were compared using Fisher’s exact tests, and continuous variables were compared using nonparametric Mann-Whitney U-tests. Statistical significance was set at p < 0.05.

Characteristics		Surgery group (n = 10)			Drainage group (n = 15)			p-value
			Range	SD		Range	SD	
Age	Years (median)	65.5	50-100	16.1	76	40-92	13.2	0.26
Gender	Male (%)	7 (70.0)	-		12 (80.0)	-		0.65
	Female (%)	3 (30.0)			3 (20.0)			
Smoking habit	Pack-years	20	5-40.5	9.9	20 (0-60)	0-60	19.7	0.92
Diabetes	No (%)	6 (60.0)	-		12 (80.0)	-		0.38
	Yes (%)	4 (40.0)			3 (20.0)			
Use of oral anticoagulant agents	No (%)	10 (100.0)	-		11 (73.3)	-		0.13
	Yes (%)	0 (0.0)			4 (26.7)			
Performance status	＜2 (%)	8 (80.0)	-		12 (80.0)	-		1
	2≦ (%)	2 (20.0)			3 (20.0)			
Body mass index	kg/m^2^ (median)	22.7	17.0-30.9	5.2	20.6	13.7-30.6	5.1	0.29
Duration of drainage	Days (median)	11	7-55	14.2	8	1-33	9.7	0.54
Hospital stay	Days (median)	27.5	13-65	13.9	35	10-98	25.3	0.1
Duration of antimicrobial therapy	Days (median)	24.5	21-82	21.4	43	23-89	18.1	0.08
Interval to post-treatment CT imaging	Days (median)	167.5	99-398	112	342	92-2958	780.8	0.16
Lung volume before treatment	mL (median)	2283	1341-3557	683.8	2277	1750-3555	642.5	0.82
Lung volume after treatment	mL (median)	3770	2693-5072	828.6	3528	2085-5288	790.9	0.41
Lung collapse rate	% (median)	32	5-64	17.8	34	3-69	17.9	0.82
Lung improvement rate	% (median)	82	67-93	9.1	68	46-78	9.3	0.003

**Figure 2:**
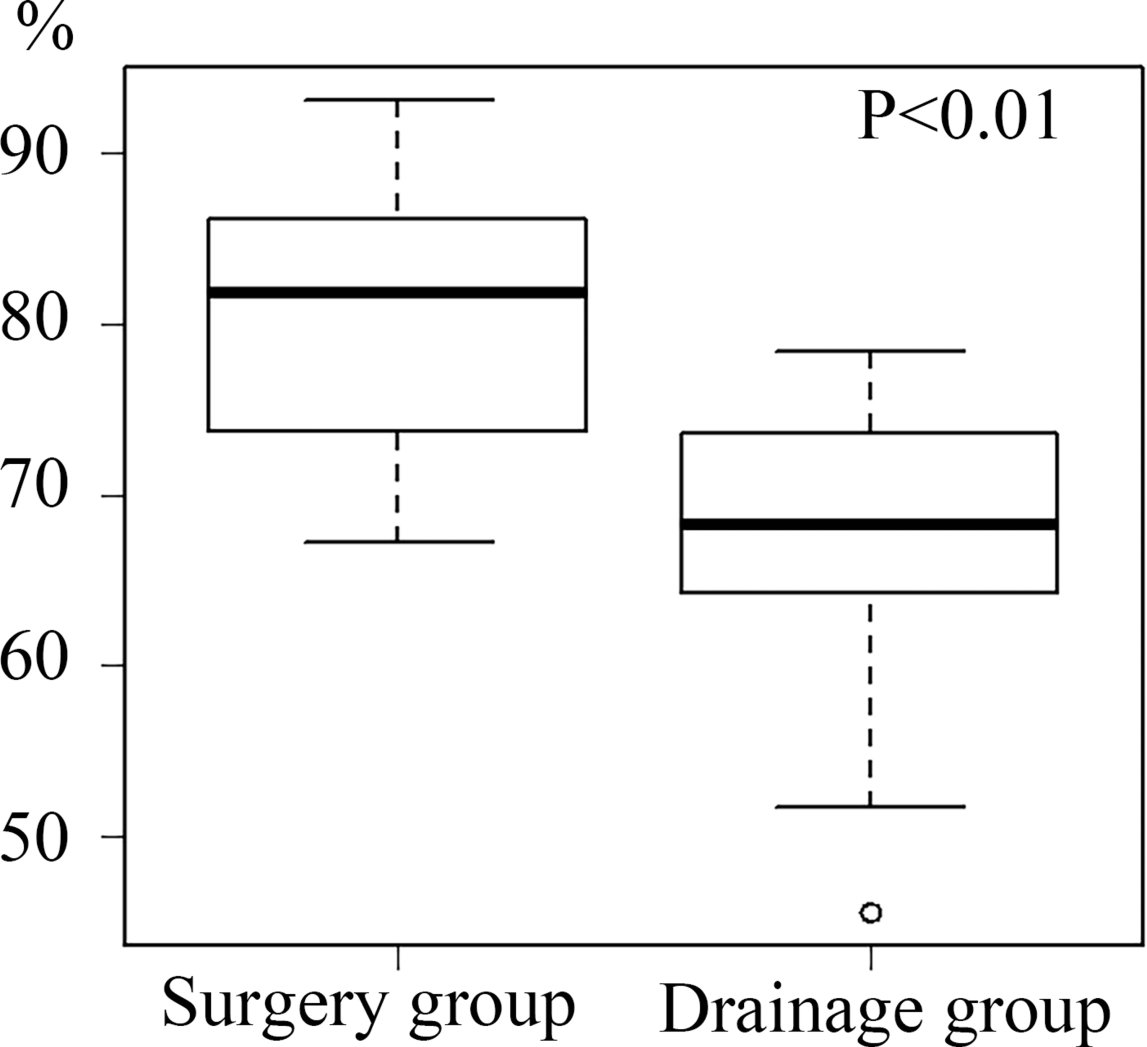
Comparison of pulmonary improvement rate (all cases) Box-and-whisker plots showing lung improvement rates after treatment. The boxes represent the interquartile range (IQR; 25th to 75th percentiles), and the horizontal line within each box indicates the median. Whiskers extend to the minimum and maximum values within 1.5× IQR from the quartiles. Individual dots represent outliers. Statistical significance was assessed using the Mann-Whitney U test (p < 0.05). Pulmonary improvement was significantly greater in the surgery group compared to the drainage group (82% vs. 68%, p < 0.01).

In the surgery group, one patient underwent surgery first due to difficulty with drainage. The remaining nine patients underwent surgery due to poor drainage and a lack of clinical improvement. The median time from drainage to surgery was seven days (range: 0-36 days). Surgery was performed using open thoracotomy in one case and thoracoscopy in the other. The patient who underwent open thoracotomy showed a lung improvement rate of 73.7%, which was lower than the median recovery rate of 85% observed in the nine patients treated with thoracoscopy. All 10 patients underwent curettage, and partial decortication was performed in three cases. Pulmonary improvement was significantly greater in the surgery group compared to the drainage group (82% vs. 68%; p < 0.01) (Figure 2).

**Figure 3:**
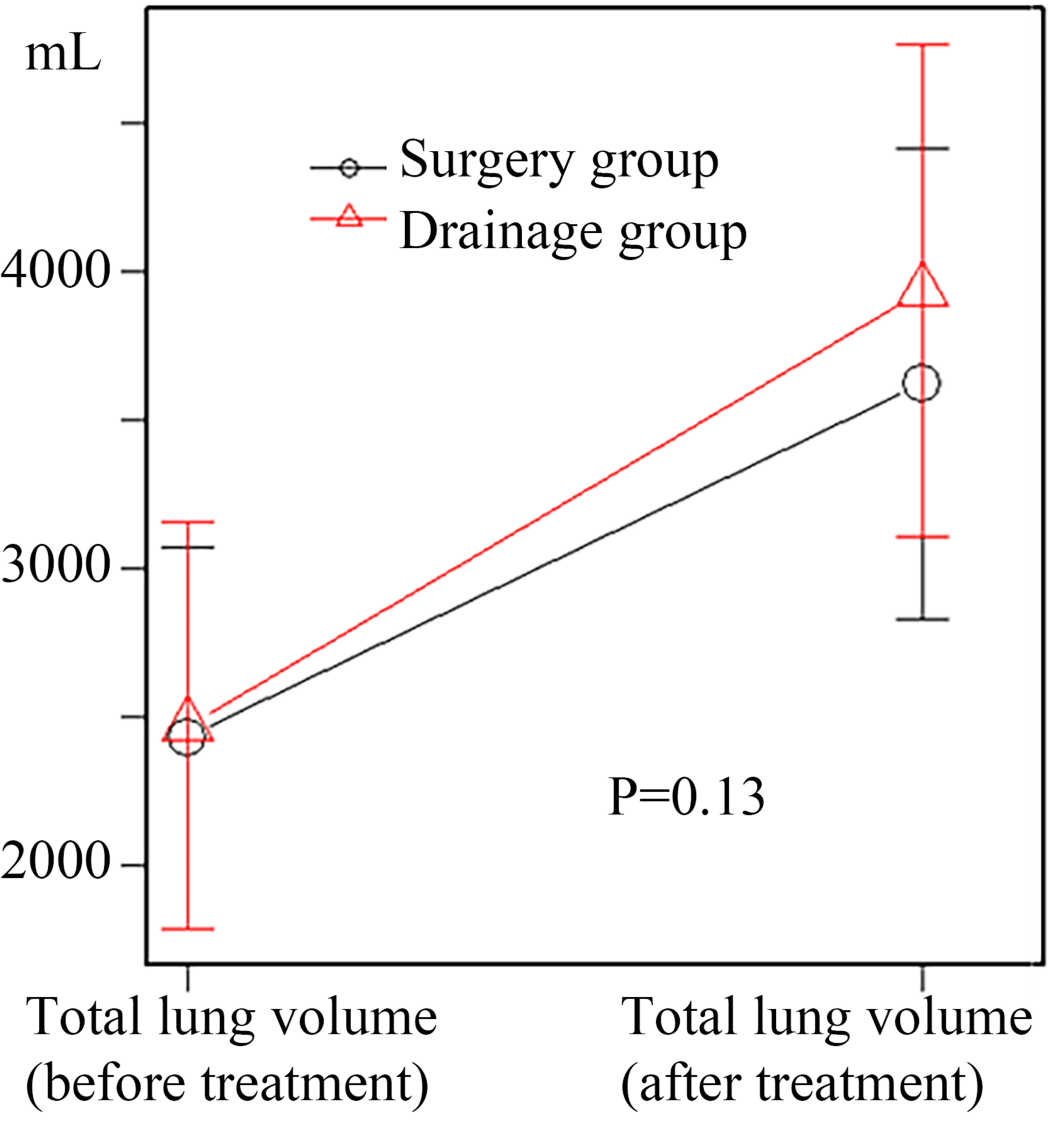
Total lung volume changes pre- and post-treatment (all cases) In the repeated measures analysis of variance, no statistically significant change in total lung volume was observed before and after empyema treatment in the overall cohort; nonetheless, a trend toward improvement was noted in the surgery group (p = 0.13).

While no statistically significant difference was observed in the change in total lung volume pre- and post-treatment, the surgery group tended to show greater improvement (Figure 3).

The duration of drainage did not differ significantly between the groups. Similarly, there was no significant difference in the length of hospital stay between the drainage group and the surgery group (27.5 vs. 35 days; p = 0.1). The duration of antimicrobial therapy also showed no significant difference between the groups (24.5 vs. 43 days; p = 0.08). The interval between treatment and post-treatment CT imaging was generally shorter in the surgery group than in the drainage group (167.5 vs. 342 days; p = 0.16).

Factors demonstrating p < 0.3 were age, BMI, and whether or not anticoagulation was performed. These three objective variables were used to perform logistic regression to assess the presence or absence of surgery (Table 2).

**Table 2:** Logistic regression results for propensity score estimation Because the use of oral anticoagulants was observed exclusively in one treatment group, leading to complete separation in the logistic regression model for propensity score estimation, these cases were excluded from the propensity score matching.

Predictor variable	Coefficient	Odds ratio	95% confidence interval	p-value
Intercept	-0.297	0.74	0.00-574.00	0.93
Use of oral anticoagulant agents	-18.3	0	0.00-infinity	1
Body mass index	0.047	1.05	0.88-1.25	0.6
Age	-0.012	0.99	0.93-1.05	0.69

A propensity score was generated and matched between the groups. After matching, nine patients were selected for each group (surgery and drainage) (Table 3).

**Table 3:** Characteristics of the two treatment groups after propensity score matching CT: computed tomography; SD: standard deviation Categorical variables were compared using Fisher’s exact tests, and continuous variables were compared using nonparametric Mann-Whitney U-tests. Statistical significance was set at p < 0.05.

Characteristics		Surgery group (n = 9)			Drainage group (n = 9)			p-value
			Range	SD		Range	SD	
Age	Years (median)	68	54-100	15.3	73	40-87	15	0.93
Gender	Male (%)	7 (77.8)	-		8 (88.9)	-		1
	Female (%)	2 (22.2)			1 (11.1)			
Smoking habit	Pack-years	20	10-40.5	8.5	20	0-60	20	0.79
Diabetes	No (%)	5 (55.6)	-		6 (66.7)	-		1
	Yes (%)	4 (44.4)			3 (33.3)			
Use of oral anticoagulant agents	No (%)	9 (100.0)	-		9 (100.0)	-		-
	Yes (%)	0 (0)			0 (0)			
Performance status	＜2 (%)	7 (77.8)	-		9 (100.0)	-		0.47
	2≦ (%)	2 (22.2)			0 (0.0)			
Body mass index	kg/m^2^ (median)	24.4	17-30.9	5.4	21.3	17.7-30.6	5.2	0.76
Duration of drainage	Days (median)	12	6-55	14.9	8	19-1	6.6	0.35
Hospital stay	Days (median)	28	20-65	13.4	28	10-76	19.7	1
Duration of antimicrobial therapy	Days (median)	25	21-82	22.4	41	23-60	13	0.22
Interval to post-treatment CT imaging	Days (median)	138	99-398	123.4	342	92-2958	968.3	0.48
Lung volume before treatment	mL (median)	2397	1341-3557	696.4	2277	1750-3555	703.2	0.895
Lung volume after treatment	mL (median)	3836	2693-5072	854.3	3503	2085-5288	965.5	0.31
Lung collapse rate	% (median)	32	22-64	20	34	3-69	15.4	0.83
Lung improvement rate	% (median)	79	67-93	9.5	66	46-78	11	0.012

**Figure 4:**
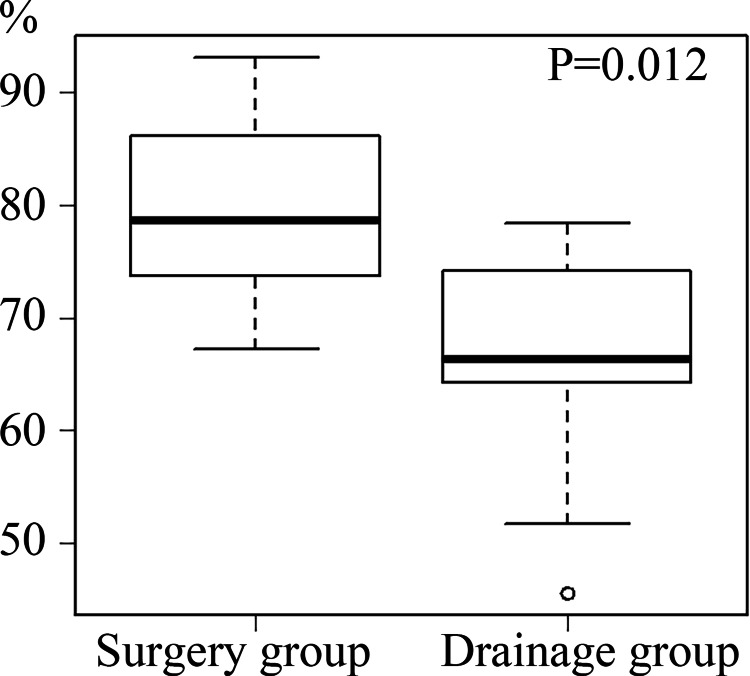
Comparison of lung improvement rate (matched cases) Box-and-whisker plots showing lung improvement rates after treatment. The boxes represent the interquartile range (IQR; 25th to 75th percentiles), and the horizontal line within each box indicates the median. Whiskers extend to the minimum and maximum values within 1.5× IQR from the quartiles. Individual dots represent outliers. Statistical significance was assessed using the Mann-Whitney U test (p < 0.05). Pulmonary improvement rate was significantly better in the surgery group (79% vs. 66%, p = 0.012).

No significant differences or trends were observed in patient background. The pulmonary improvement rate was significantly better in the surgery group (Figure 4).

**Figure 5:**
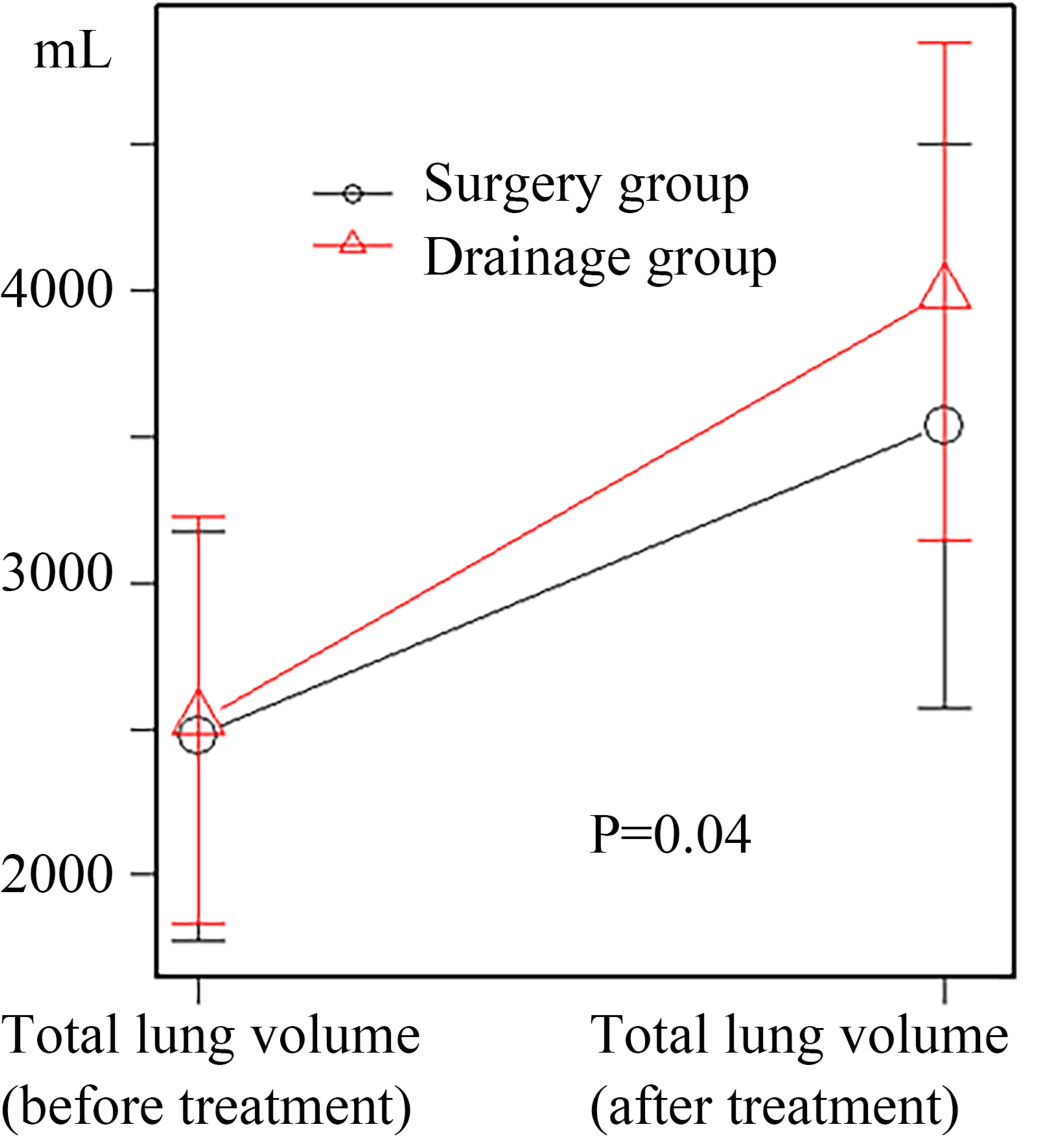
Pre- and post-treatment changes in total lung volume (matched cases) Repeated measures analysis in the matched cohort demonstrated that the surgery group exhibited a significantly greater change in total lung volume pre- and post-treatment (p = 0.012).

No significant differences in the duration of drainage and hospital stay were observed, but a trend toward shorter antimicrobial therapy duration was noted in the surgery group (25 vs. 41 days; p = 0.22). A statistically significant time-dependent improvement in total lung volume was observed in the surgery group following treatment (Figure 5).

## Discussion

The study investigated whether surgical treatment improves post-treatment lung expansion in patients with empyema. After propensity score matching, the surgery group had a 13% higher lung improvement rate compared to the drainage group. While this value did not meet the initially predefined threshold, it is nonetheless considered both statistically and clinically significant. Additionally, the comparison of total lung volume pre- and post-treatment, using repeated measures analysis of variance, revealed a significant post-treatment increase in total lung volume in both groups, in both the overall cases and the matched cases. In the overall cases, no significant difference was found in the magnitude of increase between the two groups. However, in the matched cases, the increase was significantly greater in the surgery group. In terms of median values, the improvement in total lung volume was 1,439 mL in the surgery group and 1,225 mL in the drainage group. This corresponds to a 17.5% greater improvement in the surgery group compared to the drainage group, which is consistent with the findings from another analysis.

In Stage 2, the empyema cavity is typically loculated, and re-expansion of the lung is often not achievable with chest drainage alone [14]. In actual surgical practice, extensive pleural adhesions between the lung and chest wall are frequently observed, and even after adhesiolysis, re-adhesion tends to occur within a short period of time. In one study, pulmonary segmentectomy followed by postoperative pleurodesis was reported to decrease percent vital capacity (%VC) by approximately 5.5% [15]. In cases of acute empyema, similar or even more severe pleural adhesions are likely to develop. Although surgical intervention may not necessarily reduce the extent of pleural adhesion formation compared to drainage alone, it may facilitate early re-expansion of the lung, thereby preventing adhesion in a collapsed state and potentially contributing to improved postoperative pulmonary function.

With regard to the secondary outcomes, contrary to previous reports [4-6], no significant improvement was noted in the surgery group. Surgical intervention is generally reported to shorten both drainage duration and hospital stay by approximately four to nine days [4,5]. In the present study, surgery was performed at a median of seven days after the initiation of treatment, with a median drainage duration of four days prior to surgery. This delay in surgical intervention may have diminished the potential benefits of surgery reported in previous studies, particularly in terms of reducing drainage and hospitalization periods.

In this study, we considered that bias would exist with respect to the selection of eligible patients and the indication for surgery. Eligible patients should have undergone a CT scan after a certain period of time following treatment. However, some patients who died due to unsuccessful empyema treatment or were transferred to a convalescent hospital due to disuse syndrome or other reasons after treatment could not undergo CT scans; in these instances, CT scans could not be acquired. Thus, this study is limited to patients with a good clinical course of treatment after acute empyema. However, in this study, there were no postoperative deaths during the follow-up period in the surgery group, and no cases were excluded for reasons other than CT abnormalities in the unaffected lung. Therefore, only the drainage group consisted exclusively of cases with favorable courses. This was considered to introduce a bias that was, in fact, disadvantageous to the surgery group. In the evaluated cases, the timing of post-treatment CT imaging was determined by the attending physicians and varied considerably. Although the optimal timing for imaging has not been clearly established, CT scans performed within one to two months after treatment often failed to demonstrate full resolution. Therefore, follow-up imaging should ideally be conducted after a sufficiently long interval. In this study, patients with CT abnormalities in the unaffected lung were excluded due to their potential to significantly influence image analysis outcomes. These cases may pose inherent limitations when assessing treatment-related improvements via CT.

Moreover, we hypothesized the presence of a selection bias in the decision-making process of the physician or surgeon to recommend the surgery. To minimize this bias, this study investigated patient characteristics that may have influenced the decision to perform surgery. Considering that the number of cases was small and, thus, finding significant differences would be unlikely, p < 0.3 was used as the cutoff value for item selection with possible bias. The results indicate that surgery was not performed in the anticoagulation cases, and that elderly patients and patients with low BMIs who were potentially infertile also tended to avoid the procedure. Patients who were receiving oral anticoagulants were excluded from the propensity score analysis due to complete separation. Therefore, the findings may not be generalizable to populations with higher thromboembolic risk or anticoagulant use. Furthermore, none of the cases in this study received adjunctive fibrinolytic therapy following chest drainage. The incorporation of such therapy may reduce the need for surgical intervention and could have a favorable impact on post-treatment pulmonary recovery in the drainage group [16]. Although we believe that bias was eliminated to some extent by propensity score matching, we consider that a larger study would be desirable.

It has been noted that in many cases of Stage 2 acute empyema, areas corresponding to Stage 3 may already be present [9]. As mentioned previously, in the surgical group of this study, there was a median delay of seven days from the initial intervention to surgical treatment, except in one case. Since the empyema was diagnosed as Stage 2 at the time of initial drainage, it is possible that this delay allowed further progression, resulting in an increased presence of Stage 3 lesions by the time of surgery. Although this progression likely had a negative impact on pulmonary recovery in the surgical group, the outcomes still surpassed those of the drainage group. Furthermore, surgical intervention for acute empyema performed more than 21 days after onset has been reported to be associated with a higher incidence of complications [17]. From all these perspectives, early surgical intervention should be considered when a diagnosis of Stage 2 acute empyema is made. In addition, the effectiveness of pulmonary decortication has been demonstrated in cases of chronic empyema [10,11]. Therefore, we considered that in cases closer to Stage 3, the addition of decortication may lead to greater improvement in lung re-expansion after treatment.

## Conclusions

This retrospective study indicates that surgical intervention for Stage 2 acute empyema may lead to greater improvement in lung re-expansion compared to chest drainage alone, particularly when evaluated using CT-derived lung volume as a surrogate for pulmonary function. Despite inherent limitations, such as selection bias and delays in surgical timing due to the retrospective nature of the study and clinical decision-making processes, propensity score matching enabled a more balanced comparison between groups. These findings underscore the potential clinical advantage of surgical intervention in the management of Stage 2 empyema.
